# Bronchiectasis in patients hospitalized with acute exacerbation of COPD in Spain: Influence on mortality, hospital stay, and hospital costs (2006-2014) according to gender

**DOI:** 10.1371/journal.pone.0211222

**Published:** 2019-01-25

**Authors:** Gema Sánchez-Muñoz, Ana Lopez-de-Andrés, Valentín Hernández-Barrera, Rodrigo Jiménez-García, Fernando Pedraza-Serrano, Luis Puente-Maestu, Javier de Miguel-Díez

**Affiliations:** 1 Respiratory Department, Hospital General Universitario Gregorio Marañón, Facultad de Medicina, Universidad Complutense de Madrid (UCM), Instituto de Investigación Sanitaria Gregorio Marañón (IiSGM), Madrid, Spain; 2 Preventive Medicine and Public Health Teaching and Research Unit. Health Sciences Faculty. Rey Juan Carlos University, Alcorcón, Madrid, Spain; University of Notre Dame Australia, AUSTRALIA

## Abstract

**Purpose:**

The objectives of this study were to analyze the characteristics of male and female patients hospitalized with acute exacerbation of chronic obstructive pulmonary disease (AE-COPD) during 2006–2014 according to the presence or absence of bronchiectasis and to study the factors associated with in-hospital mortality (IHM) in patients hospitalized with AE-COPD and concomitant bronchiectasis.

**Methods:**

We used the Spanish National Hospital Database to analyze patients admitted with AE-COPD as their primary diagnosis. Patients included in the study were stratified according to the presence or absence of bronchiectasis as their secondary diagnosis.

**Results:**

We identified 386,646 admissions for AE-COPD, of which 19,679 (5.09%) involved patients with concomitant bronchiectasis. When patients with and without bronchiectasis were compared, we observed that the incidence of infection by *Pseudomonas aeruginosa* was substantially higher in the former, as were the mean stay, cost, and percentage of readmissions, although IHM and comorbidity were lower. The course of patients with AE-COPD and bronchiectasis was characterized by a gradual increase in prevalence and mean age among men and no differences in prevalence or lower mean age in women. Mortality was 4.24% and 5.02% in patients with and without bronchiectasis, respectively, although significance was lost after a multivariate adjustment (OR 0.94; 95% CI, 0.88–1.01). The factors associated with IHM were older age, higher comorbidity, isolation of *P*. *aeruginosa*, mechanical ventilation and readmission.

**Conclusions:**

The prevalence of admission with AE-COPD and bronchiectasis increased in men but not in women during the study period. In patients hospitalized with AE-COPD, we did not find differences in mortality when comparing the presence and absence of bronchiectasis. The analysis of temporal trends revealed a significant reduction in mortality from 2006 to 2014 in male patients with COPD and concomitant bronchiectasis, but not among women. It is important to consider the factors associated with IHM such as age, comorbidity, isolation of *P*. *aeruginosa*, mechanical ventilation and readmission to better identify those patients who are at greater risk of dying during hospitalization.

## Introduction

Chronic obstructive pulmonary disease (COPD) and bronchiectasis are two of the most common diseases of the airway [1,2]. The prevalence of COPD in the general population is approximately 10%, although many cases go undiagnosed [1]. The disease has high rates of morbidity and mortality and is a major public health problem [2]. The prevalence of bronchiectasis varies significantly between countries [3–5], with values ranging from 52.3 cases per 100,000 adults in the USA [3] to 2.7 per 100,000 persons in Finland [4] to 3.7 per 100,000 persons in the pediatric population of New Zealand [5]. COPD represents a major health problem [6].

Patients with COPD and bronchiectasis have specific clinical characteristics, such as greater sputum production, a greater degree of dyspnea, and a higher number of exacerbations. Notably, Spanish guidelines on COPD (Ges-EPOC) propose computed tomography (CT) in patients with the “exacerbated with chronic bronchitis” phenotype to rule out bronchiectasis because this condition has specific implications for therapy [7]. In turn, prognosis is worse for patients with both diseases. Thus, the presence of bronchiectasis is associated with greater mortality in patients with COPD independently of the degree of lung function involvement, the BODE index, and the number of exacerbations [8].

Between 5% and 10% of patients with bronchiectasis also have COPD [9–11]. Furthermore, the prevalence of bronchiectasis in patients with moderate or severe COPD ranges from 4% to 72% depending on the series. In any case, this prevalence is greater than that recorded for the general population and increases with the severity of COPD. The above data support an association between both processes that is more than merely random. More in-depth analysis is necessary to clarify this association [12].

Data collection on patients hospitalized with COPD and bronchiectasis at the national level is important when evaluating the incidence, clinical characteristics, morbidity, mean stay, cost, and mortality of this association. In Spain, current national registries of patients with respiratory diseases such as bronchiectasis could provide more data on the association [13]. However, the number of patients with COPD and concomitant bronchiectasis remains very low [14].

The objectives of this study were as follows: a) To analyze the characteristics of men and women hospitalized with acute exacerbation of COPD (AE-COPD) during 2006–2014 according to the presence or absence of bronchiectasis and b) To study the factors associated with in-hospital mortality (IHM) between patients hospitalized with AE-COPD and concomitant bronchiectasis.

## Material and methods

On discharging a hospitalized patient in the Spanish Health System, physicians must declare all diagnoses made and procedures performed using the codes of the International Classification of Disease, 9th Revision (ICD-9-CM). This information is collected in the Spanish National Hospital Database (“Conjunto Minimo Basico de Datos” [CMBD]), which compiles data from all hospitals in the Spanish National Health System [15]. The CMBD includes personal data (sex and date of birth), date of admission, date of discharge, discharge destination (home, deceased, or other health care/social care institution), and details of up to 14 discharge diagnoses and up to 20 procedures performed during admission.

We selected all admissions of patients with exacerbation of COPD (ICD-9-CM code 491.21) as their primary diagnosis during 3 periods of 3 years each between 2006 and 2014. We stratified the database according to the presence of bronchiectasis (ICD-9-CM code 494) as a secondary diagnosis.

We analyzed the prevalence, clinical characteristics, diagnostic procedures, and outcomes of men and women discharged after an exacerbation of COPD according to whether they also had bronchiectasis. Clinical characteristics included information on overall comorbidity at the time of hospitalization, which was graded using the Charlson comorbidity index (CCI) [16]. The CCI comprises 17 disease categories whose scores are totaled to obtain an overall score for each patient. The proportion of patients who died during admission (IHM), the length of hospital stay, and the costs were also estimated for each study period. The costs were calculated using diagnosis-related groups for the disease. These groups represent a medical cost entity applicable to a set of diseases requiring analogous management resources [17]. All costs were adjusted for inflation in Spain during the study period.

We also analyzed the variable “Readmission”. According to the Spanish National Hospital Database methodology, a “readmission” is defined as a patient that was discharged from a hospital and then readmitted to that hospital within the last month. The variable is considered positive for any patient discharged in the last month beside which was the primary diagnosis from the previous admission.

Finally, we studied predictors of IHM in men and women discharged after an exacerbation of COPD with concomitant bronchiectasis.

### Statistical analysis

A descriptive statistical analysis was performed. Quantitative variables were expressed as the mean (SD). Qualitative variables were expressed as frequencies and percentages. Comparisons were performed using the chi-squared test, Fisher’s exact test, *t* test, or ANOVA, as appropriate. Multivariate analysis to investigate time trends for study variables were conducted using exact logistic regression analysis for small outcomes and logistic regression for variables with sufficient outcome. Logistic regression models were used to assess factors associated with in-hospital mortality.

To build the multivariable regression models, we performed the following process:

1. We conducted bivariate analysis for each study variable to assess its association with the dependent variable.
2. Selection of variables for the multivariable analysis. We included those variables with a significant association in the bivariate tests and other study variables that were found to be important by other authors in the references reviewed, even if their values were not significant.
3. To decide which independent variables remained in the final model, we used the Wald statistic after including them one by one. Consecutive models were created as new variables were included. These consecutive models were compared with the previous models using the likelihood ratio test.
4. Once the final model was found, we checked for the presence of collinearity and interactions between variables in the model. We limited interactions to first order (two by two) interactions. None of the collinearity or interaction analyses showed a significant association.

Estimates were made using STATA version 10.1 (StataCorp LP, College Station, Texas, USA). Statistical significance was set at a 2-tailed alpha of 0.05.

### Ethical aspects

Full data confidentiality was observed according to Spanish legislation. Patient identifiers were removed before the database was provided to the authors to maintain strict patient confidentiality. It was not possible to identify individual patients in this study or in the database. Given the anonymous and mandatory nature of the dataset, it was not necessary to obtain informed consent. The Spanish Ministry of Health evaluated our study protocol, concluded that it fulfilled the ethical requirements of Spanish legislation, and provided us with the anonymous database. Ethical approval was not necessary for the above reasons.

## Results

We identified a total of 386,646 admissions with AE-COPD as the main diagnosis in Spain between 2006 and 2014. Of these, 19,679 patients (5.09%) had bronchiectasis as a secondary diagnosis. Patients with COPD and bronchiectasis included 16,903 men (85.89%) and 2,776 women (14.11%).

### Characteristics of the study population

Table 1 shows the characteristics of patients admitted with AE-COPD according to the presence or absence of associated bronchiectasis. Men were predominant in both groups. The mean age was higher in the patients with concomitant bronchiectasis than in those without (75.31 ± 9.15 vs. 74.72 ± 10.48 years, respectively, p<0.001). In contrast, the percentage of smokers was lower in the first group of patients (43.37% vs. 46.95% respectively, p<0.001). Comorbidity was also significantly lower in the patients with COPD and bronchiectasis (for ≥ 2 points, 21.92% vs. 27.83% respectively, p<0.001). The same occurred with obesity (5.63% vs. 10.61%, respectively, p<0.001).

**Table 1:** Characteristics of patients discharged after a COPD exacerbation according to the presence of bronchiectasis in Spain from 2006 to 2014.

		Bronchiectasis		
		No (n = 366967)	Yes (n = 19679)	
	Category	n (%)	n (%)	p-value
Gender	Men	313645(85.47)	16903(85.89)	0.100
	Women	53322(14.53)	2776(14.11)	
Age groups	<65 years	58901(16.05)	2421(12.3)	<0.001
	65–79 years	176377(48.06)	10289(52.28)	
	≥80 years	131689(35.89)	6969(35.41)	
Charlson comorbidity index	0	156806(42.73)	9889(50.25)	<0.001
	1	104712(28.53)	5476(27.83)	
	≥ 2	105449(28.74)	4314(21.92)	
Current tobacco use	Yes	172284(46.95)	8535(43.37)	<0.001
Obesity	Yes	38927(10.61)	1108(5.63)	<0.001
Pseudomonas aeruginosa infection	Yes	9198(2.51)	2310(11.74)	<0.001
S Pneumoniae infection	Yes	1086(0.3)	61(0.31)	0.724
Legionella infection	Yes	12(0)	1(0.01)	0.669
S. Aureus infection	Yes	96(0.03)	4(0.02)	0.620
H. Influenzae infection	Yes	57(0.02)	3(0.02)	0.975
Virus infection	Yes	231(0.06)	5(0.03)	0.038
Aspergilosis infection	Yes	960(0.26)	156(0.79)	<0.001
Thoracic computed tomography	Yes	24973(6.81)	2756(14)	<0.001
Bronchoscopy	Yes	3555(0.97)	341(1.73)	<0.001
Invasive mechanical ventilation	Yes	3450(0.94)	97(0.49)	<0.001
Non-invasive mechanical ventilation	Yes	19691(5.37)	983(5)	0.024
Eosinophilia	Yes	115(0.03)	7(0.04)	0.745
Aerosol therapy	Yes	78220(21.32)	3890(19.77)	<0.001
Oxygen therapy	Yes	104566(28.49)	5800(29.47)	0.003
In hospital mortality	Yes	18436(5.02)	835(4.24)	<0.001
Readmission	Yes	63232(17.23)	4405(22.38)	<0.001
Age (years), mean (SD)		74.72(10.48)	75.31(9.15)	<0.001
Length of stay (days), mean (SD)		8.27(7.77)	9.57(8.48)	<0.001
Cost (euros), mean (SD)		3664.75(2928.41)	3971.67(2191.80)	<0.001

P value for comparison between those with and without bronchiectasis

As for microorganisms isolated (Table 1), the proportion of patients infected by *Pseudomonas aeruginosa* was significantly greater among those who had associated bronchiectasis than those who did not (11.74% vs. 2.51%, respectively, p<0.001). The results were similar for *Aspergillus* species (0.79% vs. 0.26%, respectively, p<0.001).

As for the procedures, CT and bronchoscopy were performed in more patients with bronchiectasis than in those without (14% and 1.73% vs. 6.81% and 0.97%, respectively, p<0.001). Furthermore, the percentage of patients who required invasive mechanical ventilation was significantly lower among those with bronchiectasis than those without (0.49% vs. 0.94%, respectively, p<0.001). The use of noninvasive mechanical ventilation was also significantly lower among patients with bronchiectasis (p = 0.024). However, oxygen therapy was more frequent in patients with concomitant bronchiectasis than in those who did not have the disease (29.47% vs. 28.49%, p = 0.003). In contrast, nebulizers were less frequent in the first group (19.77% vs. 21.32%, respectively, p<0.001).

The mean length of hospital stay was greater in patients with bronchiectasis (9.57 vs. 8.27 days, respectively, p<0.001). The same was found for the cost and proportion of readmissions (€3,971.67 vs. €3,664.75 and 22.38% vs. 17.23%, respectively, p<0.001 in both cases). In contrast, in-hospital mortality was lower in patients with COPD and bronchiectasis than in those without bronchiectasis (4.24% vs. 5.02%, respectively, p<0.001).

### Characteristics of the population suffering from COPD exacerbation and bronchiectasis according to gender

**Fig 1:**
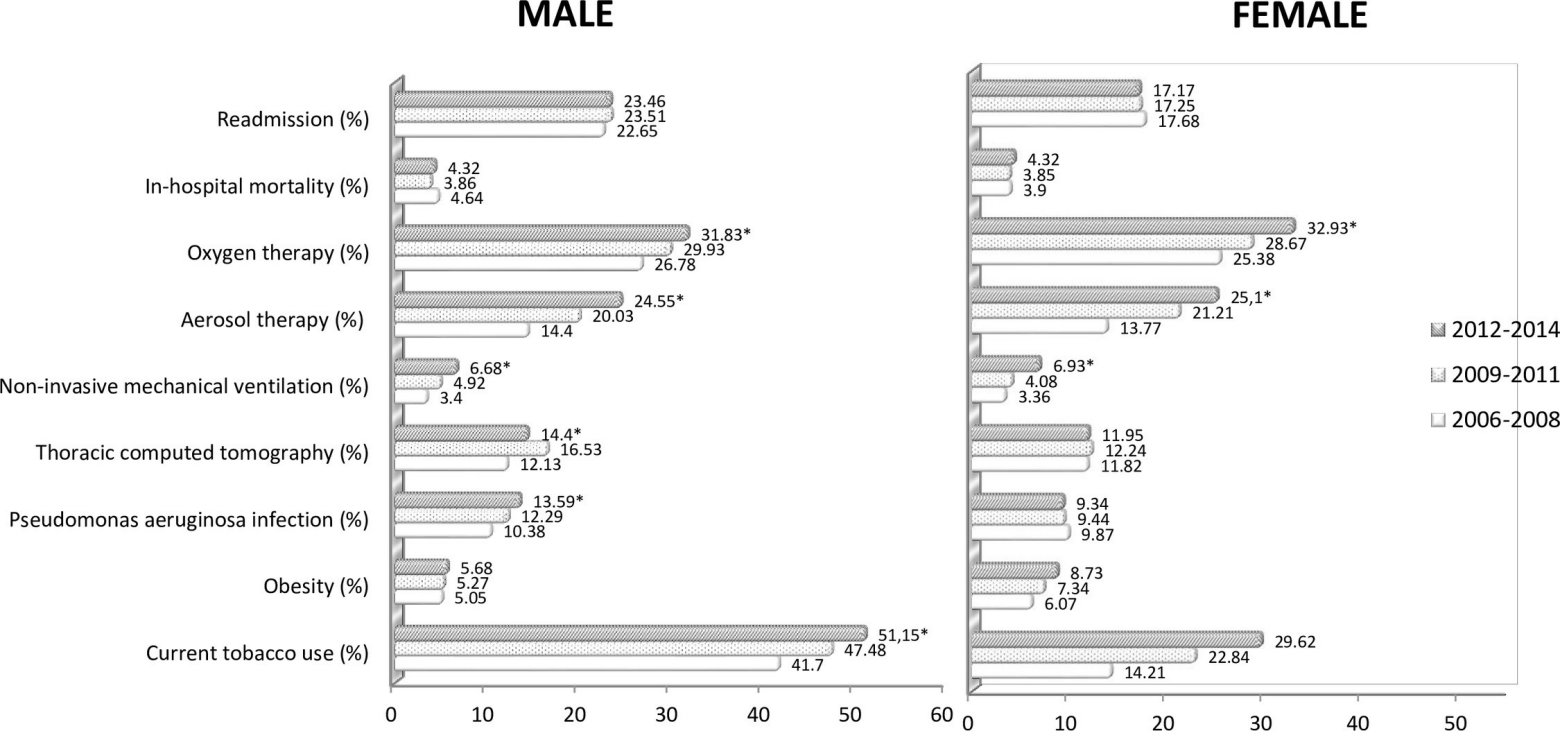
Readmission rates, in hospital mortality, diagnosis and therapeutic procedures, *P aeruginosa* infection, obesity and current tobacco use among men and women discharged after a COPD exacerbation and who suffered from concomitant bronchiectasis in Spain from 2006 to 2014. **Footnote:** * Significant time trend (p<0.05). Exact logistic regression analysis has been used to estimate the time trend for variables with small outcome. Logistic regression for variables with sufficient outcome. In both cases the models were adjusted by for age, sex, and other covariates as appropriate.

Fig 1 and Table 2 show the prevalence, clinical features, diagnosis procedures and outcomes on men and women discharged after a COPD exacerbation and who suffered from concomitant bronchiectasis in Spain from 2006 to 2014.

**Table 2:** Prevalence, age distribution, comorbidity, microorganisms, diagnosis and therapeutic procedures, eosinophilia, length of stay and cost of men and women discharged after a COPD exacerbation and suffering concomitant bronchiectasis in Spain from 2006 to 2014.

		MALE				FEMALE			
		2006–2008	2009–2011	2012–2014	OR (95%CI) p-value	2006–2008	2009–2011	2012–2014	OR (95%CI) p-value
Prevalence, n (%)		5624(4.88)	5486(5.1)	5793(5.37)	1.05(1.03–1.07) <0.001^a^	922(5.03)	858(4.85)	996(4.97)	0.99(0.95–1.04) 0.885 ^a^
Age (years), mean (SD)		74.57(9.22)	75.23(9.17)	76.07(8.89)	<0.001	75.24(10.97.)	76(10.97)	75(11.09)	<0.001
Age groups, n (%)	< 65 years	740(13.16)	664(12.1)	582(10.05)	0.86(0.81–0.93) <0.001 ^a^	122(13.23)	130(15.15)	183(18.37)	1.21(1.07–1.38) 0.002 ^a^
	65–79 years	3184(56.61)	2919(53.21)	2965(51.18)	0.89(0.86–0.93) <0.001 ^a^	464(50.33)	360(41.96)	397(39.86)	0.81(0.74–0.89) <0.001 ^a^
	≥80 years	1700(30.23)	1903(34.69)	2246(38.77)	1.21(1.16–1.26) <0.001 ^a^	336(36.44)	368(42.89)	416(41.77)	1.11(1.04–1.22) 0.020 ^a^
Charlson comorbidity index, n (%)	None	3073(54.64)	2740(49.95)	2593(44.76)	0.83(0.80–0.86) <0.001 ^a^	515(55.86)	475(55.36)	493(49.5)	0.87(0.79–0.95) 0.005 ^a^
	One	1485(26.4)	1518(27.67)	1672(28.86)	1.06(1.01–1.10) 0.003 ^a^	264(28.63)	254(29.6)	283(28.41)	0.99(0.90–1.10) 0.906 ^a^
	Two or more	1066(18.95)	1228(22.38)	1528(26.38)	1.21(1.16–1.27) <0.001 ^a^	143(15.51)	129(15.03)	220(22.09)	1.27(1.12–1.43) <0.001 ^a^
S Pneumoniae infection, n(%)	Yes	23(0.41)	22(0.4)	8(0.14)	0.64(0.42–0.87) 0.011 ^a^	2(0.22)	4(0.47)	2(0.2)	0.96(0.48–1.94) 0.998 ^b^
Legionella infection, n (%)	Yes	1(0.02)	0(0)	0(0)	0.61(0.00–5.69) 0.665 ^b^	0(0)	0(0)	0(0)	NA
S. Aureus infection, n (%)	Yes	1(0.02)	1(0.02)	1(0.02)	0.98(0.17–5.54) 0.999 ^b^	0(0)	0(0)	1(0.1)	1.48(0.16-inf) 0.717 ^b^
H. Influenzae infection, n (%)	Yes	2(0.04)	0(0)	0(0)	0.32(0.00–1.84) 0.221 ^b^	1(0.11)	0(0)	0(0)	0.62(0.00–5.60) 0.664 ^b^
Virus infection, n (%)	Yes	0(0)	3(0.05)	2(0.03)	1.85(0.53–9.01) 0.439 ^b^	0(0)	0(0)	0(0)	NA
Aspergilosis infection, n (%)	Yes	40(0.71)	49(0.89)	52(0.9)	1.14(0.93–1.40) 0.276 ^a^	8(0.87)	5(0.58)	2(0.2)	0.52(0.26–1.03) 0.105 ^b^
Bronchoscopy, n (%)	Yes	103(1.83)	102(1.86)	114(1.97)	1.03(0.90–1.18) 0.752 ^a^	6(0.65)	8(0.93)	8(0.8)	1.09(0.67–1.78) 0.722 ^b^
Invasive mechanical ventilation, n (%)	Yes	41(0.73)	17(0.31)	24(0.41)	0.72(0.54–0.96) 0.048 ^a^	8(0.87)	4(0.47)	3(0.3)	0.58(0.30–1.12) 0.103 ^b^
Eosinophilia, n (%)	Yes	2(0.04)	3(0.05)	1(0.02)	0.77(0.33–1.78) 0.784 ^a^	0(0)	0(0)	1(0.1)	1.48(0.16-inf) 0.718 ^b^
Readmission	Yes	1274(22.65)	1290(23.51)	1359(23.46)	1.02(0.91–1.16) 0.482 ^a^	163(17.68)	148(17.25)	171(17.17)	0.98(0.89–1.11) 0.952 ^a^
Length of stay (days), mean (SD)		10.29(9.49)	9.45(8.00)	9.07(8.02)	<0.001	10.21(8.06)	9.32(9.07)	8.77(6.97)	<0.001
Cost (euros), mean (SD)		3931.3(1737.2)	3866.7(2368.2)	3813.4(2358.4)	<0.001	4013.1(2649.7)	3873(2854.9)	3769.2(1034.9)	0.023

P value and Odds Ratios with 95% confidence intervals (OR 95%CI) for time trend.

^a^ Exact logistic regression analysis has been used to estimate the time trend for variables with small outcome.

^b^ Logistic regression for variables with sufficient outcome. In both cases time trend models were adjusted by for age, sex, and other covariates as appropriate. For continues variables we used ANOVA and only the P value for the significance is shown.

In men, the prevalence of bronchiectasis increased over time from 4.88% in 2006–2008 to 5.37% in 2012–2014 (p<0.001). No statistically significant temporal variations were found for women.

The percentage of smokers increased gradually in both sexes, from 41.7% in men and 14.21% in women during 2006–2008 to 51.15% and 29.62%, respectively, in 2012–2014 (p<0.001 in both cases). Comorbidity also increased significantly over time in both sexes (percentage of patients with ≥ 2 comorbid conditions: 18.95% in men and 15.51% in women during the first period; 26.38% in men and 22.09% in women during the second period; p<0.001 in both cases).

As for isolation of the microorganism, the frequency of infection by *P*. *aeruginosa* increased significantly in men (from 10.38% in 2006–2008 to 13.59% in 2012–2014, p<0.001). In contrast, the frequency of isolation of pneumococcus decreased over time in men (from 0.41% in 2006–2008 to 0.14% in 2012–2014, p = 0.011).

As for diagnostic procedures, the percentage of chest CT scans increased over time in men (from 12.13% in the first time period to 14.4% in the third, p<0.001). The need for invasive mechanical ventilation decreased gradually in men (from 0.73% in 2006–2008 to 0.41% in 2012–2014, p = 0.048), whereas the use of noninvasive mechanical ventilation increased (from 3.4% to 6.68%, p<0.001). The frequency of oxygen therapy and aerosol therapy increased significantly for both sexes over time.

IHM and readmissions remained stable in both sexes during the study period. The mean length of hospital stay decreased significantly in both men and women (from 10.29 and 10.21 days, respectively, in 2006–2008 to 9.07 and 8.77 days, respectively, in 2012–2014, p<0.001). The mean hospital cost decreased significantly over time from €3,931.3 to €3,813.4 in men and from €4,013.1 to €3,769.2 in women.

### Predictors of IHM in patients with COPD and concomitant bronchiectasis

**Fig 2:**
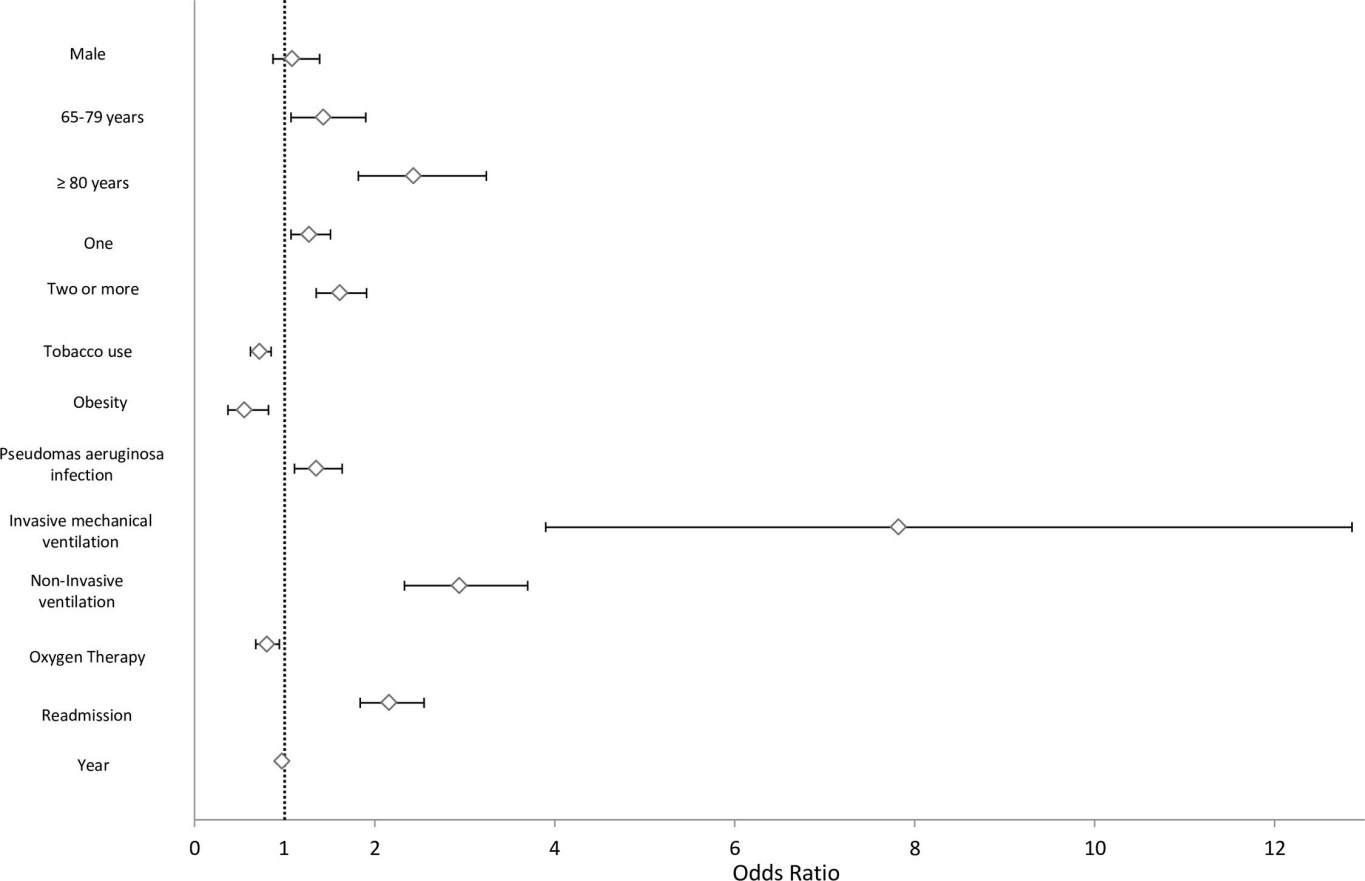
Predictors of in hospital mortality among patients discharged after a COPD exacerbation and suffering concomitant bronchiectasis in Spain from 2006–2014. Footnote: The variables shown are those that remained in the final multivariable model.

Fig 2 shows the factors associated with IHM in patients with COPD and concomitant bronchiectasis. After multivariable adjustment, the IHM is not different between women and men (OR 1.08 [95% CI 0.87–1.39]). Predictors of higher IHM include age >65 years, comorbidity, infection by *P*. *aeruginosa* (OR, 1.35 [95% CI 1.11–1.64]), and readmission (OR, 2.16 [95% CI, 1.86–2.50]).Another strong predictor of IHM was mechanical ventilation, both invasive (OR, 7.82 [95% CI, 4.75–12.85]) and noninvasive (OR 2.94 [95% CI, 2.33–3.70]). Oxygen therapy and current tobacco use were protective factors.

The multivariable analysis of temporal trends revealed a significant reduction in mortality over time in patients with COPD and concomitant bronchiectasis (OR, 0.97 [95% CI, 0.94–0.99]).

The results of the factors associated with IHM in patients with COPD and concomitant bronchiectasis by gender are shown in S1 Table. For men, all variables that yielded significant results in the entire population showed significant OR. For women, only invasive and noninvasive ventilation increased the IHM, and current tobacco use was a protective factor.

Finally, using the entire database including patients with and without bronchiectasis, we found that the presence of bronchiectasis was not associated with IHM after adjusting for age, sex and CCI as possible confounders (OR 0.94 [95% CI, 0.88–1.01]).

## Discussion

We found that 5.09% of patients admitted with AE-COPD had concomitant bronchiectasis. This frequency was higher in men and older persons. Previous studies report very different frequencies [18,19]. Such wide variability can be explained, at least in part, by differences in study methodology, patient characteristics, and diagnostic criteria. In the meta-analysis of Yingmeng et al [20], in which the prevalence of bronchiectasis in individuals with COPD was 54.3%, the patients had to have been diagnosed with COPD according to the GOLD criteria [21], and the diagnosis of bronchiectasis had to be confirmed using CT. As in our study, persons with COPD and bronchiectasis were older and more often men. We also recorded lower comorbidity in patients with concomitant bronchiectasis, in contrast to reports from other studies, where an association was found between the presence of bronchiectasis in patients with COPD and a greater number of comorbid conditions [22].

As for the microorganisms isolated, we found a greater proportion of infections by *P*. *aeruginosa* and *Aspergillus* species in patients with AE-COPD and bronchiectasis. The association between the presence of potentially pathogenic microorganisms such as *P*. *aeruginosa* and bronchiectasis in patients with COPD has been confirmed in previous studies [20, 23–26]. Furthermore, while *P*. *aeruginosa* isolation increased, pneumococcal isolation decreased in men discharged after an AE-COPD who suffered from concomitant bronchiectasis. Among the causes that could justify these results are a more frequent search for *P*. *aeruginosa* or more frequent use of pneumococcal vaccination over time in this group of patients.

Diagnostic test results showed that more CT scans and bronchoscopies were performed in patients with bronchiectasis. The potential explanations for this finding are as follows: first, CT is the method of choice for diagnosing this disease; and, second, hemoptysis or infectious processes that require collection of respiratory samples are more likely in these patients than in those with no concomitant bronchiectasis.

We found no differences in the use of noninvasive mechanical ventilation in the 2 groups of patients evaluated; however, invasive ventilation was more frequently used in AE-COPD patients without bronchiectasis. Greater age and poorer lung function in patients with AE-COPD and bronchiectasis may explain the fact that noninvasive ventilation is established as the ceiling of therapy in some cases, thus ruling out more aggressive management. Nevertheless, although admission to the intensive care unit is unusual in patients with bronchiectasis, it is becoming increasingly frequent, as shown by Navaratnam et al [27].

Cost, length of hospital stay, and number of readmissions were higher in COPD patients with bronchiectasis than in those who did not have bronchiectasis. De la Rosa et al also found higher costs in patients with COPD and associated bronchiectasis [28]. In any case, few studies have analyzed the financial cost of bronchiectasis, although available data appear to indicate that it is a major health care expense [29,30].

We found that crude in-hospital mortality was significantly lower in patients with AE-COPD and bronchiectasis, although the differences disappeared after the multivariate adjustment. Previous studies showed that the risk of death in COPD increased significantly in the presence of bronchiectasis [8,24,25]. Given the greater risk involved, patients with COPD and bronchiectasis may seek medical care earlier, may be admitted to the hospital with less severe disease, and may receive more care from physicians, thus accounting for our findings. However, consistent with our findings, no association was recorded between the presence of bronchiectasis and mortality in a study of 406 patients who had COPD and severe exacerbations [22] or a study with 338 patients who had no previous exacerbations [31].

Our study showed that the vast majority (>85%) of exacerbations occurred among men, although other studies have reported that women can be more susceptible to exacerbations than men [32]. This gender discrepancy has also been described by other authors [19]. Furthermore, we observed various differences according to gender. For men, there was a gradual increase over time in the prevalence of bronchiectasis, mean age, comorbidity, number of chest CT scans performed, and percentage of isolations of *P*. *aeruginosa*. Comorbidity also increased over time in women. In contrast, the mean age tended to decrease in women, and no significant differences were observed for the prevalence of admissions, the use of CT scans and bronchoscopy, or the percentage of isolations of *P*. *aeruginosa*. These findings could be explained by the lower number of women evaluated; therefore, it seems reasonable to perform additional studies with larger numbers of women to draw conclusions on this point. Furthermore, we observed an increase in the use of noninvasive mechanical ventilation, oxygen therapy, and aerosol therapy over time.

The mean length of hospital stay and cost decreased during the study period in both men and women with AE-COPD and concomitant bronchiectasis, as recorded in other studies on patients with bronchiectasis [33]. We also found a decrease in in-hospital mortality and the number of readmissions, as reported elsewhere in patients with COPD [34].

The factors associated with greater in-hospital mortality in patients with AE-COPD and concomitant bronchiectasis were age, comorbidity, isolation of *P*. *aeruginosa*, readmission, and use of mechanical ventilation (both invasive and noninvasive). Other authors have also found that the use of this therapeutic modality during hospitalization is associated with an increased risk of mortality in patients with acute exacerbations of bronchiectasis [35]. In contrast, smoking was a protective factor, likely because the more severely ill patients or those with more advanced disease are more likely to quit smoking or because the diagnostic coding was skewed. Few studies have evaluated general mortality rates in patients with bronchiectasis [36,37]. Roberts et al [37] reported a 3% annual gradual increase in mortality due to bronchiectasis in England and Wales during 2001–2007. In contrast, our study between 2006 and 2014 recorded a reduction in mortality over time, thus potentially indicating that the management of these patients has been improving over time.

The main strength of our study lies in its large sample size and standardized methodology. The Spanish National Hospital Database methodology has not changed since it was implemented in 1987. Nevertheless, our study is subject to limitations. First, the use of ICD-9-CM diagnostic codes to identify patients hospitalized for COPD and bronchiectasis constitutes a potential source of bias in terms of the quality of data and extrapolation to reality. Given that we cannot verify that COPD was diagnosed based on spirometry or that all diagnoses of bronchiectasis were confirmed by CT, both diagnoses could be under- or overestimated. Notably, less than 10% of patients had a chest CT during their admission, which would have confirmed or excluded a diagnosis of bronchiectasis. Some patients may have already had bronchiectasis diagnosed on CT prior to their admission, but the low prevalence of CT orders (especially among patients “with bronchiectasis”) remains a concern regarding diagnosis accuracy. Furthermore, since the results are limited to the coded variables, relevant data may not be included, such as the severity of airflow obstruction and the treatment or diagnosis procedures received. It is logical to think that the management of hospitalized patients for AE-COPD has changed and improved over the study period as more modern diagnosis methods and treatments become available. However, Ginde et conducted a study to assess the validity of ICD 9 CM codes to identify AE-COPD in the emergency department finding that code 491.2x proved to be effective to identify most cases [38].

Despite these limitations, the CMBD has the advantage of being mandated by the Spanish National Health System and that it covers nearly 100% of admissions in Spain [39]. In addition, Spain is a large country with a public health system that provides full medical coverage free of charge to the entire population; therefore, patients come from a variety of socioeconomic categories, thus improving the external validity of our results.

## Conclusions

The prevalence of admission with AE-COPD and bronchiectasis increased in men but not in women during the study period. We did not find differences in mortality in patients hospitalized with AE-COPD depending on the presence of absence of bronchiectasis. The analysis of temporal trends revealed a significant reduction in mortality from 2006 to 2014 in patients with COPD and concomitant bronchiectasis. It is important to consider the factors associated with IHM such as age, comorbidity, isolation of *P*. *aeruginosa*, mechanical ventilation and readmission to better identify those patients who are at greater risk of dying during hospitalization.

Additional studies are necessary to better explain the differences found between men and women.

## Supporting information

S1 Table Predictors of in hospital mortality among men and woman discharged after a COPD exacerbation and suffering concomitant bronchiectasis in Spain from 2006–2014. The variables shown are those that remained in the final multivariable model. VNIFM variable not included in the final model.
(DOCX)

## Abbreviations

AE-COPD: exacerbation of chronic obstructive pulmonary disease
CCI: Charlson comorbidity index
CMBD: Spanish National Hospital Database (“Conjunto Minimo Basico de Datos”)
CT: computed tomography
ICD-9-CM: International Classification of Disease, 9th Revision
IHM: in-hospital mortality
