# Supplementary material for: Bronchiectasis in patients hospitalized with acute exacerbation of COPD in Spain: Influence on mortality, hospital stay, and hospital costs (2006-2014) according to gender
Source: PLoS One. 2019 Jan 25;14(1):e0211222. doi: 10.1371/journal.pone.0211222 (PMC6347366; doi:10.1371/journal.pone.0211222)
Supplement: S1 Table — The variables shown are those that remained in the final multivariable model. VNIFM variable not included in the final model. (DOCX) [file pone.0211222.s001.docx]

**Supplementary Table 1. Predictors of in hospital mortality among men and woman discharged after a COPD exacerbation and suffering concomitant bronchiectasis in Spain from 2006-2014.**

|  |  | **MALE** | **FEMALE** |
| --- | --- | --- | --- |
|  |  | OR (CI 95%) | OR (CI 95%) |
| Age groups | <65 years | 1 | 1 |
|  | 65-79 years | 1.57(1.14-2.15) | 0.80(0.40-1.63) |
|  | ≥80 years | 2.60(1.89-3.58) | 1.64(0.82-3.29) |
| Charlson comorbidity index | None | 1 | VNIFM |
|  | One | 1.28(1.07-1.55) | VNIFM |
|  | Two or more | 1.65(1.37-1.98) | VNIFM |
| Current tobacco use | No | 1 | 1 |
|  | Yes | 0.74(0.63-0.87) | 0.41(0.21-0.82) |
| Obesity | No | 1 | 1 |
|  | Yes | 0.57(0.37-0.88) | VNIFM |
| Pseudomonas aeruginosa infection | No | 1 | VNIFM |
|  | Yes | 1.33(1.09-1.65) | VNIFM |
| Invasive mechanical ventilation | No | 1 | 1 |
|  | Yes | 6.59(3.76-11.56) | 8.75(1.14-27.30) |
| Non-invasive mechanical ventilation | No | 1 | 1 |
|  | Yes | 3.07(2.40-3.92) | 2.32(1.17-4.60) |
| Oxygen therapy | No | 1 | VNIFM |
|  | Yes | 0.82(0.69-0.97) | VNIFM |
| Readmission | No | 1 | VNIFM |
|  | Yes | 2.28(1.96-2.67) | VNIFM |
| Year | | 0.96(0.93-0.99) | 1.02(0.95-1.10) |

The variables shown are those that remained in the final multivariable model.

VNIFM variable not included in the final model
